# CMR stress testing in a patient with morbid obesity (BMI 58 kg/m^2^) and suspected coronary artery disease

**DOI:** 10.1186/s12872-018-0779-3

**Published:** 2018-03-05

**Authors:** Lukas Stoiber, Bernhard Schnackenburg, Rolf Gebker, Hanane Hireche-Chikaoui, Burkert Pieske, Sebastian Kelle

**Affiliations:** 1 German Heart Center Berlin, Department of Internal Medicine/Cardiology, Berlin, Germany; 2 Charité Campus Virchow Klinikum, Department of Internal Medicine/Cardiology, Berlin, Germany; 3 DZHK (German Centre for Cardiovascular Research), Partner Site Berlin, Berlin, Germany; 4 Philips Health Care, Hamburg, Germany

**Keywords:** Obesity, Stress test, CMR, Cardiac magnetic resonance tomography, Coronary artery disease

## Abstract

**Background:**

Severe obesity is asscociated with an increased risk of coronary artery disease (CAD) but non-invasive cardiac imaging modalities have important technical limits.

**Case presentation:**

We report a case of a 58-year old patient with suspected CAD and severely elevated BMI of 58 kg/m^2^.

**Conclusions:**

Stress-CMR was able to non-invasively stratify risk with good imaging quality despite the body dimensions of the patient.

## Background

Severe obesity is defined as a body mass index (BMI) ≥40 kg/m^2^ and is independently asscociated with an increased risk of coronary artery disease (CAD) [1–3]. Despite common use in the general population, conventional imaging modalities as stress-echo have important technical limits for overweight individuals. In nuclear medecine, some stress/rest perfusion protocols provide high image quality [4]. However, limitations for tracer uptakes in obese patients undergoing ^123^I–metaiodobenzylguanidine (MIBG)-scintygraphy, widely used for risk stratification in heart failure, have been reported [5]. Cardiac magnetic resonance (CMR) imaging perfusion, also known as stress-CMR, is not depending on acoustic windows and has been also reported to be safe and feasible in obese patients [2, 6, 7]. In addition, CMR is able to perform perfusion, viability and ventricular function within a single examination [7].

## Case presentation

We are reporting a case of a 58-year old woman with a BMI of 58,08 kg/m^2^ who was referred for stress-CMR to our department. She had undergone ambulatory stress-echo but image quality was poor as a result of body habitus. No treadmill exercise test could be performed. She was reporting exertional dyspnea of NYHA class II and no history of chest pain, syncope or palpitations. Due to the patient’s extreme body weight and concomitant diabetes suspicion for CAD was raised. She did not use any medication and had no fevers, night sweats, upper respiratory symptoms, cough, nausea, vomiting, diarrhea, rash, arthralgias or urinary tract symptoms and no history of asthma or allergy to contrast agents. Blood pressure at admission was 149/79 mmHg, heart rate 75 beats per minute at rest. The 12-lead ECG showed sinus rhythm with slightly notched S in the inferior leads but no signs of cardiac hypertrophy. Laboratory-test prior to admission found elevated liver enzymes. Medical history included cholecystectomy several years ago. Abdominal ultrasound had shown nonalcoholic fatty liver disease (NAFLD) supposingly as a result of abnormal metabolism. Cholesterol levels where slightly elevated, hemoglobin A1c level of 8.2% indicated poor glycemic control.

**Fig. 1:**
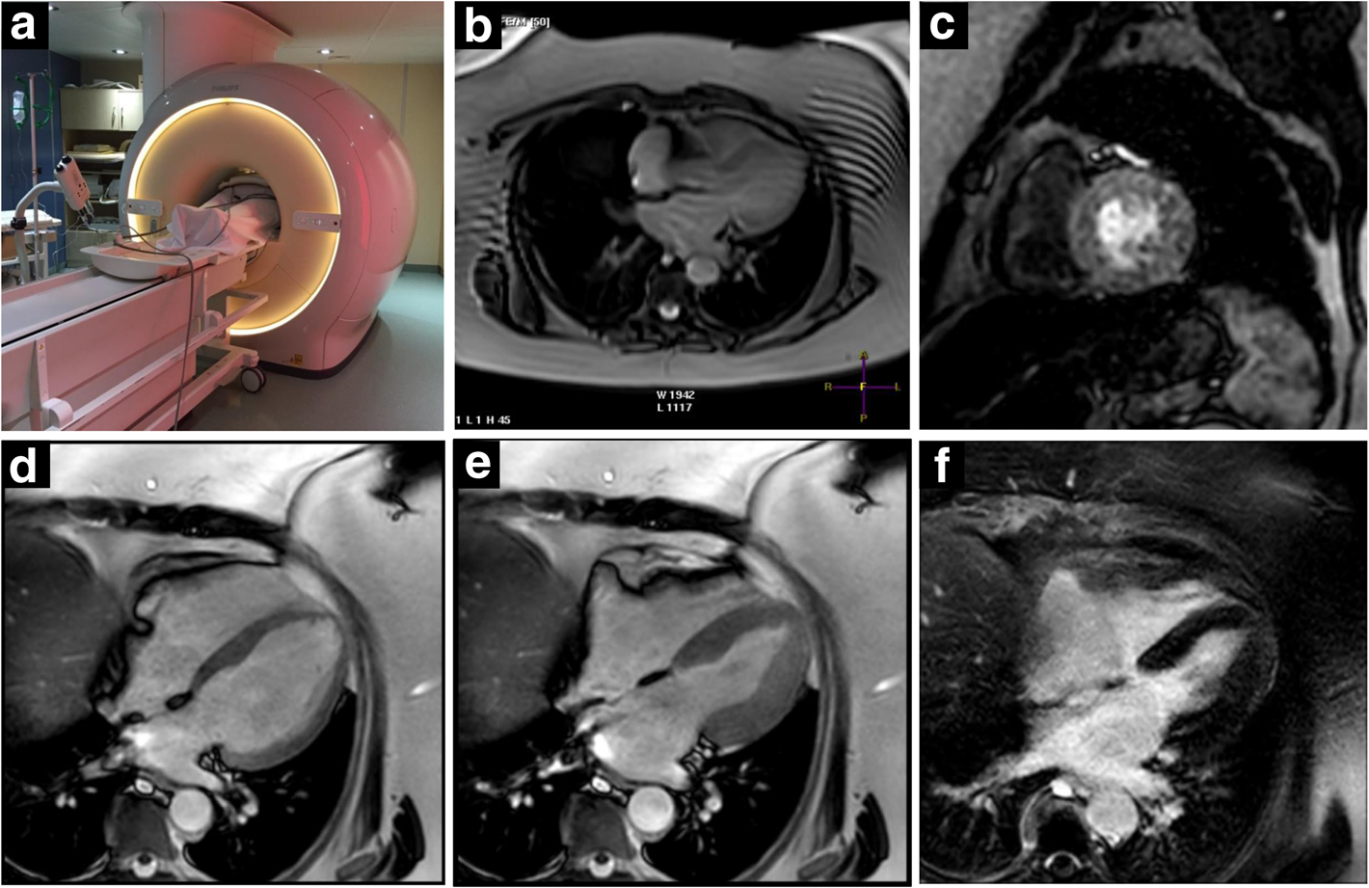
**a** Philips Ingenia 3.0 Tesla Scanner with 70 cm wide bore and patient inside. **b** Survey showing severe obesity and some artefacts out of the field of interest. **c** Mid short axes of vasodilator stress perfusion CMR revealed no ischemia, with excellent image quality. **d-e** Four-chamber double oblique SSFP images at end-diastole (d) and end-systole (e) reveal excellent endocardial border detection for quantification of LV volumes, function and mass. **f** Late gadolinium enhancement post contrast administration was able to exclude myocardial fibrosis or scar; image quality was excellent

**Fig. 2:**
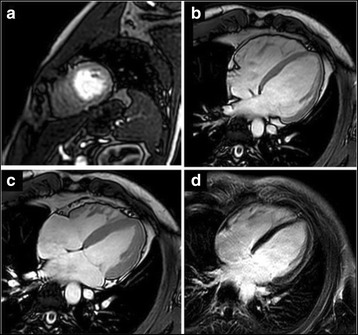
CMR images of a patient with normal body weight (BMI 24 kg/m^2^). **a** Mid short axes of vasodilator stress perfusion CMR revealed no ischemia **b-c** Four-chamber double oblique SSFP images at end-diastole (**b**) and end-systole (**c**) reveal excellent endocardial border detection for quantification of LV volumes, function and mass. **d** Late gadolinium enhancement post contrast administration without findings of myocardial scar or fibrosis

**Fig. 3:**
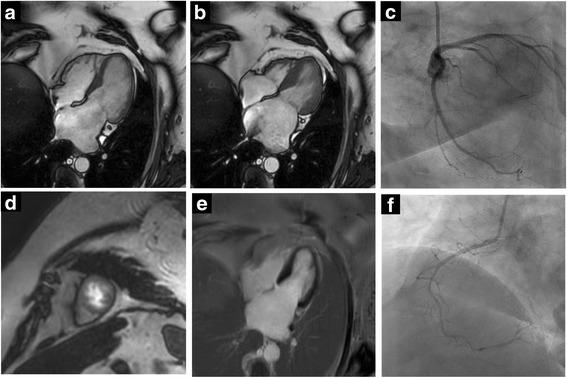
CMR images of a patient with obesity (BMI 37 kg/m^2^). **a-b** Four-chamber double oblique SSFP images at end-diastole (**a**) and end-systole (**b**) reveal excellent endocardial border detection for quantification of LV volumes, function and mass. **d** Apical short axes of vasodilator stress perfusion CMR revealed no ischemia. **e** Late gadolinium enhancement post contrast administration without findings of myocardial scar or fibrosis. Invasive catheterization excluded hemodynamic significant epicardial coronary artery stenosis at left anterior descending artery and left circumflex artery (**c**) as well as right coronary artery (**f**)

We performed a stress-CMR on a Philips Ingenia 3.0 Tesla Scanner with a 70 cm wide bore system using phased array receive coils (16 elements anterior, 12 elements posterior) which allowed examination despite the body dimensions of the patient (Fig. 1A). Cine imaging with SSFP, perfusion and delayed hyperenhancement (LGE) protocol were started. Image acquisition was performed without any notable complications, the patient intermittently reported mild forms of chest pain and dypnea. The horizontal long axis (HLA), vertical long axis (VLA), and short axis (SAX) planes (Field of View 250 mm (x) /250 mm (y); Pixel spacing 1.12 mm (x) /1.12 mm (y); Flip angle 45°; Echo time 1,75 ms; Repetition time 3,5 ms; Image resolution 224 × 224 pixels) demonstrated preserved biventricular function with a LV-EF of 65% and normal ventricular and atrial dimensions. Mild concentric hypertrophy was seen, with a basal septum enddiastolic diameter of 12 mm. Regadenoson in a dose of 400 μg was administered as a vasodilatory agent, the total gadolinium dose was 22 mmol. Perfusion-images showed high diagnostic image quality throughout the protocol (Fig. 1C), comparable to perfusion-sequences in a normal-weight and obese individuals (Figs. 2 and 3). No exercised perfusion deficits indicating myocardial ischemia were detected in either one of the three acquired short-axis views and there was no sign of myocardial scar tissue in the LGE images (Fig. 1F).

## Conclusions

Overall, stress-CMR was feasible and absence of ischemia or myocardial scar was documented in the examination. Due to the high negative predictive value of a negative stress-CMR, there was no need for further invasive cardiac examination [8]. Stress-CMR was able to non-invasively stratify risk with good imaging quality in a patient with suspected CAD and severely elevated BMI.

## Abbreviations

BMI: Body mass index
CAD: Coronary artery disease
CMR: Cardiac magnetic resonance
HLA: Horizontal long axis
MIBG: ^123^I–metaiodobenzylguanidine
LGE: Late gadolinium enhancement
NAFLD: Nonalcoholic fatty liver disease
NYHA: New York Heart Failure Association
SAX: Short axis
SSFP: Steady-state free precession
VLA: Vertical long axis
